# “A letter for Dr. Outgroup”: on the effects of an indicator of competence and chances for altruism toward a member of a stigmatized out-group

**DOI:** 10.3389/fpsyg.2015.01422

**Published:** 2015-09-25

**Authors:** Jens H. Hellmann, Anne Berthold, Jonas H. Rees, Deborah F. Hellmann

**Affiliations:** ^1^Center of Teaching in Higher Education, University of MünsterMünster, Germany; ^2^Department of Psychology, University of ZurichZurich, Switzerland; ^3^Department of Social Psychology and Center for Interdisciplinary Research, University of BielefeldBielefeld, Germany; ^4^Criminological Research Institute of Lower SaxonyHannover, Germany

**Keywords:** altruism, prosocial behavior, discrimination buffer, competence, lost-letter technique

## Abstract

The lost letter technique is an unobtrusive method to investigate attitudes in a particular population. Ostensibly lost letters from senders who apparently belong to different groups or addressed to recipients from apparently different groups are dispersed in public places, and return rates represent a measure of altruistic or discriminatory behavior toward one group or another. In two field experiments using the lost letter technique, we investigated the influence of group membership and the presence or absence of a doctorate degree as an indicator of competence on the likelihood of receiving helping behavior. Experiment 1 showed that a generic member of a low-status ethnic out-group (Turks living in Germany) was the target of discrimination, while a generic member of a non-stigmatized out-group (French in Germany) was not. Moreover, when the name of the member from the stigmatized out-group was (vs. was not) preceded by a doctorate degree, more of the allegedly lost letters were returned. There were no such differential effects for recipients who were members of the in-group (Germans) or the non-stigmatized out-group (French). Experiment 2 showed that a recipient from the stigmatized out-group (Turk) with a doctorate degree received more letters when the sender was German versus Turkish (i.e., from the recipient’s own group). Overall, the sender’s ethnic group membership was an important factor for the likelihood of receiving an ostensibly lost letter, in that fewer letters arrived from a sender with a Turkish (vs. German) name. We conclude that the likelihood of altruistic behavior toward out-group members can increase when in-group members intend to communicate with competent out-group members. Therefore, under certain conditions, the presentation of a highly competent member of an otherwise stigmatized out-group may serve as a discrimination buffer.

## Introduction

The investigation and analysis of conflicts between groups has been among the core interests of many, if not all, social sciences since their inception. This is particularly true for social psychology where studies on the dynamics of prejudice and intergroup conflict have arguably been the discipline’s single most defining research topic over many decades (e.g., Allport, 1954; Sherif et al., 1961). A host of classic studies document reliably that individuals tend to treat members of their in-group more favorably than out-group members (Tajfel et al., 1971; Mullen et al., 1992), and various theories build on this in-group preference to explain intergroup conflict from different perspectives such as the *Social Identity Theory* (SIT; Tajfel and Turner, 1979) or the *Realistic Intergroup Conflict* perspective (RIC; e.g., Sherif et al., 1961). This *in-group favoritism* or *in-group bias* has been found in many different domains such as the assignment of more positive traits to an in-group (vs. out-group) member (Cadinu and Rothbart, 1996). Particularly in the domain of helping behavior, the group membership of a person has been identified as a crucial determinant of the likelihood of providing and receiving help: Belonging to a common group increases help for individuals (see Flippen et al., 1996; Levine et al., 2005).

More recently, research has set out to study how such differential treatment of in-group and out-group members may have evolved in humans in the first place (Choi and Bowles, 2007; for reviews see De Dreu et al., 2014; Rusch, 2014). Drawing on Darwin’s basic notion that behaviors benefiting the in-group and harming the out-group should have co-evolved, studies on such *parochial altruism* have used different paradigms and approaches. Prominent studies have used complex mathematical models to gauge the evolutionary advantage of different patterns of behaviors such as mutually beneficial, selfish, spiteful, or altruistic behaviors, where parochial altruism can be defined as a combination of altruism directed at the in-group and spite directed at the out-group (see Rusch, 2014). Following this logic, for example, García and van den Bergh (2011, p. 277) simulated different strategies in a prisoner’s dilemma situation, defining parochial altruism as instances of altruism “limited to donors and recipients belonging to the same group.” These authors found that such parochial altruism will generally be favored by selection. Given this apparent evolutionary advantage through parochial altruism, however, incidents of out-group directed altruism need to be explored further. One way of circumventing the universality of parochial altruism in humans may be to provide counter-stereotypical information about an out-group member, which can be an indicator of competence for a member of an out-group that is otherwise stigmatized as incompetent (e.g., Sinclair and Kunda, 1999).

We suggest that research on altruism may benefit from methods developed in other research areas, such as classic research on prejudice (cf. Everett et al., 2015). Mathematical modeling, idealized game theoretical situations, or even paradigms exploring the effects of neuropeptides and hormones on parochial altruism (e.g., De Dreu et al., 2010; Reimers and Diekhof, 2015) allow researchers to study the phenomenon under thoroughly controlled laboratory conditions. The present studies used an established field-setting to study altruism under less controlled but highly realistic conditions.

Field studies are particularly helpful if researchers aim to investigate helping behavior toward different groups in an unobtrusive way. Additionally, they do not exclusively rely on student samples, but more ecologically valid samples drawn from the general population. Therefore, field studies are an important instrument to explore moderators of intergroup-discrimination effects under real-world conditions. The two field experiments reported in this article were conducted in order to contribute to previous research on altruism and intergroup behavior. In a nutshell, we tested if helping behavior differs depending on the perceived competence of in-group versus out-group members using the *lost-letter technique* (Milgram et al., 1965).

In-group favoritism as one of two aspects of parochial altruism (also see Dorrough et al., 2015), when, for example, expressed through helping behavior preferably dedicated to an in-group over an out-group member, should be stronger when the out-group is stigmatized in some way than when the reference is a non-stigmatized out-group (for an overview see Penner et al., 2005). Although some conceptualizations of such differential intergroup discrimination appear to be widely accepted (e.g., Fiske et al., 2002; also see Hofstede and Bond, 1984), empirical demonstrations of differential levels of altruism toward members of out-groups with versus without the stigma of incompetence are extremely rare (for an overview regarding stigma and prejudice, see Phelan et al., 2008).

### Helping Behavior and Out-Group Member Characteristics

Groups and stereotypes can be classified by means of the Behaviors from Intergroup Affect and Stereotypes-map (BIAS, Cuddy et al., 2007). According to this approach, groups are treated differently depending on how they are perceived on the two dimensions of competence and warmth. For example, members of a less competent group are likely discriminated through *harmful* behaviors, while members of competent groups are deserving of *facilitating* behaviors. Thus, altruistic behavior such as posting a lost letter should be more probable when it is addressed toward a member of the presumably competent in-group compared to the likelihood of altruistic behavior toward a member of an out-group stigmatized as less competent. However, such differences should be less likely when comparing the in-group and a non-stigmatized out-group.

One goal of the present research was to demonstrate that out-groups are not uniformly targets of discrimination, but intergroup discrimination regarding helping behavior may vary as a function of out-group stigma. In Germany, Turks represent the largest ethnic minority out-group (see Klink and Wagner, 1999), and are generally regarded as less competent than members of the German in-group (Asbrock, 2010). In other words, the most salient stigma of Turks in Germany is their ascribed lack of competence. To cautiously foreshadow a result from our own pilot study (see below), other ethnic out-groups such as the French do not suffer from this stigma of relatively inferior competence compared to the German in-group. Accordingly, we expected differences in helping behavior between in-group and out-group to depend on the quality of stigmatization of the out-group.

While a host of research has addressed discriminatory behavior resulting from differential stereotyping; only few field studies have explored potential ways of buffering such discrimination. In a field experiment, Kaas and Manger (2012) identified one possibility of tackling intergroup discrimination on the job market: These researchers sent out application letters to German companies from applicants with a German vs. Turkish-sounding name. The typical intergroup discrimination effect, that is, more frequent interview invitations for the in-group member (also see Bertrand and Mullainathan, 2004), disappeared once the out-group member’s application included a reference letter from a former employer. Such letters of recommendation are likely the most frequently used source of finding out about a job candidate’s competence (Kaslow et al., 2007). Thus, there is some evidence that, under certain conditions, indicators of competence can moderate the intergroup discrimination effect. Another possibility to subtly implement an indicator of competence is to simply add a doctorate degree to an individual’s name (cf. Gregory, 1995; Sinclair and Kunda, 1999).

The present research also aimed at examining whether such an indicator of the group members’ competence (i.e., a doctorate degree) represents one condition, under which the discrimination effect can be attenuated or even be discontinued. In line with our above reasoning, we expected altruistic behavior in terms of posting a lost letter addressed to an ostensibly competent member of an otherwise stigmatized out-group but not to a generic member of the stigmatized out-group. In this context, the term *generic* refers to a member of the respective group who does not hold a doctorate degree.

### The Lost-Letter Technique

In the lost-letter paradigm (Milgram et al., 1965), letters are dispersed in specific areas. These letters appear to be lost by their sender. Because these fully stamped letters are basically identical and only variations in the name of the recipient or of the sender or both hints at their particular group memberships, actual intergroup discrimination can then be operationalized as the relative number of letters that are returned for each recipient or sender. When the name of the recipient on an apparently lost letter indicates a different cultural background than the sender’s name, the finder of such a letter can actively promote or impede a basic form of intergroup communication.

### The Present Research

We focused on three hypotheses: (1) We expected that a generic member of a stigmatized out-group would receive help less frequently compared to a generic in-group member. More precisely, the stigmatized out-group member should receive fewer letters than a member of the in-group. (2) An indicator of the recipient’s competence can serve as a buffer against such differences in altruistic behavior, because it may work against the stigma of the out-group. Thus, a member of a stigmatized out-group who is perceived as competent should receive more letters than a generic member of the stigmatized out-group. (3) The recipient’s competence serves as a discrimination buffer only when the *sender* is a member of the in-group. More precisely, we predicted that fewer letters would arrive for the out-group member with doctorate degree when the sender apparently belongs to the out-group (vs. in-group). We designed Experiment 1 to test Hypotheses 1 and 2, Experiment 2 was conducted to address Hypothesis 3.

The experiments were carried out in accordance with the recommendations and the approval of the Ethics Committee of the Department of Psychology and Sport and Exercise Sciences at the University of Münster, Germany.

Additionally, in a pilot study, we asked participants to indicate socially shared consensual stereotypes about the German in-group and about Turks as well as the French as out-groups. For this pilot study, we expected to find differential ratings, especially on the dimension of competence, toward the different out-group nationalities mentioned above.

## Pilot Study

In addition to previous research (Asbrock, 2010), this pilot study sought to obtain ratings of consensually shared stereotypes toward a stigmatized out-group, namely Turks, in relation to a culturally more proximate out-group, namely the French, and the German in-group.

### Method

#### Participants and Procedure

Respondents were *N* = 72 undergraduate students (56 female, 11 male, 5 did not report their gender) at the University of Münster, Germany, with a mean age of 20.68 years (*SD* = 3.56). Seven participants did not indicate their age. All participants were tested during a break within two parts of an introductory lecture on statistics. Participation took about 5 min, was completely voluntary, and was not compensated.

Participants received a single sheet of paper that constituted the questionnaire, on which they were asked to provide evaluations of different groups based on what they believed most people in Germany thought about the respective group. The instructions stressed that the study was not about the participants’ individual evaluation of the presented groups (see Asbrock, 2010).

#### Measures

Participants were asked to indicate to what extent *most people in Germany* ascribe the subsequent adjectives to the respective group. For the dimension of competence, these items were competent, competitive, and independent, for warmth, these items were likeable, warm, and good-natured. All items were assessed on a scale from 1 (*not at all*) to 5 (*completely*) and were presented in an order alternating between competence and warmth. The presented groups were Germans, Turks, and the French.

The internal consistencies of the dimensions per group were as follows: Warmth Germans, Cronbach’s α = 0.80, warmth Turks, α = 0.85, warmth French, α = 0.84, competence Germans, α = 0.67, competence Turks, α = 0.58, competence French, α = 0.74. For each of the two dimensions, competence and warmth, the corresponding three items were averaged per group so that higher values indicate higher ascriptions on the respective dimension for the respective group.

### Results

Means and standard deviations for the three groups’ scores on the two dimensions are presented in **Table 1**, which also includes comparisons between the warmth and competence ratings within each of the three groups, Germans, Turks, and the French.

**Table 1:** Pilot study: standard deviations and means of ratings of warmth and competence regarding three groups.

Group	Competence *M* (*SD*)	Warmth *M* (*SD*)	*t*	*p*	*d*
Germans	4.07 (0.54)	2.72 (0.60)	18.42	<0.001	2.18
Turks	2.54 (0.61)	2.92 (0.76)	-4.36	<0.001	-0.52
French	3.13 (0.68)	3.28 (0.89)	-1.11	0.27	-0.13

*Means and standard deviations are based on three items per dimension that were assessed using 5-point Likert-type scales. Statistical values in this table refer to tests of differences between the dimensions of competence and warmth within the respective groups.*

#### Competence

Overall, the three groups differed significantly from each other regarding ascribed competence, *F*(2,142) = 131.49, *p* < 0.001, $\eta_{p}^{2}$ = 0.65. Bonferroni-adjusted pairwise comparisons revealed that the competence ascribed to Germans was estimated as being higher than the competence ascribed to Turks, *t*(70) = 15.02, *p* < 0.001, *d* = 1.77, and to the French, *t*(70) = 10.00, *p* < 0.001, *d* = 1.19. Additionally, the competence ascribed to the French was significantly higher than the competence ascribed to Turks, *t*(70) = 6.66, *p* < 0.001, *d* = 0.79.

#### Warmth

Overall, the three groups differed significantly from each other in terms of ascribed warmth, *F*(2,142) = 11.60, *p* < 0.001, $\eta_{p}^{2}$ = 0.14. Bonferroni-adjusted pairwise comparisons revealed that the warmth ascribed to Germans did not significantly differ from the warmth ascribed to Turks, *t*(70) = -1.78, *p* = 0.24, *d* = -0.21. The French received ratings on the dimension of warmth that were higher than those for Germans, *t*(70) = 4.94, *p* < 0.001, *d* = 0.60, and for Turks, *t*(70) = 2.83, *p* = 0.02, *d* = 0.33.

### Discussion

This pilot study sought to replicate crucial aspects of previous research by Asbrock (2010) by assessing consensually shared cultural stereotypes toward the German in-group and the Turks as a stigmatized out-group. Furthermore, it was designed to extend previous research by adding a non-stigmatized out-group nationality to the list, namely the French.

Regarding warmth, the French are regarded as warmer than Germans and Turks. On this dimension, the present data did not reveal a bias in favor of the in-group nationality, which appears to be consistent with previous findings (Cuddy et al., 2009). Still, regarding the dimension of competence, Germans are seen as the relatively most competent group. Even more importantly, the Turkish out-group was clearly perceived as less competent than both the German in-group and the French out-group.

## Experiment 1

### Design and Procedure

Experiment 1 consisted of a 3 (recipient’s group: German vs. Turkish vs. French) × 2 (indicator of high competence: doctorate degree present vs. not present) design. In total, we dispersed *N* = 180 letters, *n* = 30 letters per condition, in Bremen, Germany. Consistent with the established procedure for lost-letter-studies (Milgram et al., 1965), all letters were fully stamped and included hand-written addresses for the recipient and the sender and a note on the back of each letter “found next to your car” written in German (“neben Ihrem Auto gefunden”) by a research assistant with a different pen to ensure the dispersed letters would be perceived as genuinely lost. Each letter was attached behind a car’s windshield. Previous studies with various locations of dispersion have shown that this procedure resulted in especially high return rates as compared to, for example, placing letters on the pavement (Milgram et al., 1965). The letters were shuﬄed in advance to secure random attachment per area and street. Great care was taken to make sure that no other letter was visible from the position around any car, to which another letter was attached.

The names of the ostensible recipients were Nils Schönfeld (German), Antoine Dupont (French), and Ali Yildirim (Turkish). The sender’s name on all letters was Jens Hellmann (German). Each letter contained an invitation to a birthday party, which was included in case any finder opened the letter. The content of the note could not be seen through the envelope. The sender’s address was a local address in Bremen, Germany, where the letters were distributed. The dependent variable was the number of letters per condition that arrived at the recipients’ address where a letter box displayed the names of all three recipients.

### Results and Discussion

We performed χ^2^–tests for differential return rates of the letters. Statistical tests reported for the resulting 2 × 2 contingency tables are one-tailed (see Preacher, 2001).

The return rates for in-group members (the German recipient) and members of the non-stigmatized out-group (the French recipient) were each independent of presence of doctorate degree (see **Table 2**). Importantly, however, for the stigmatized out-group (the Turkish recipient), significantly more letters arrived when a doctorate degree preceded his name (Dr. Ali Yildirim) than if no such academic title was present (Ali Yildirim), χ^2^(1, *N* = 60) = 3.35, *p* = 0.03, φ = 0.24. For the Turkish recipient without doctorate degree (Ali Yildirim), marginally fewer letters were returned than for the in-group recipient, regardless of presence of a doctorate degree of the in-group recipient (Nils Schönfeld or Dr. Nils Schönfeld, respectively), each χ^2^(1, *N* = 60) = 2.22, *p* = 0.07, φ = 0.19. This finding conceptually replicates previous field demonstrations of discrimination against stigmatized out-group members (e.g., Klink and Wagner, 1999) and is in line with previous research on parochial altruism (e.g., Choi and Bowles, 2007) in showing that altruistic helping behavior is more readily displayed for (generic) in-group members. For the French recipient (Antoine Dupont or Dr. Antoine Dupont, respectively), there were no differences in return rates, indicating that there was no general tendency to discriminate against a member of an out-group that is not generally stigmatized and no differential return rates dependent on the presence of a doctorate degree for this group.

**Table 2:** Experiment 1: return rates of letters as function of the recipient’s group membership and presence of a doctorate degree.

	Recipient’s group membership		
	In-group (German)	Stigmatized out-group (Turk)	Non-Stigmatized out-group (French)
Doctorate degree present	25 (83%)	26 (87%)	24 (80%)
Doctorate degree not present	25 (83%)	20 (67%)	24 (80%)

*Absolute number of returned letters and respective percentages in parentheses per condition. Percentages are rounded. Dispersed letters per cell were n = 30. The sender was consistently a member of the German in-group.*

To sum up, the results of Experiment 1 show that whether the German or the French recipient held a doctorate degree did not make any difference with regard to the respective return rates. When the member of the stigmatized Turkish out-group held a doctorate degree, he received significantly more letters than when the address on the letter did not include the doctorate.

However, from the results found in Experiment 1, it was not entirely clear for whom finders of a letter provided help by posting it: in fact, one could argue that finders of a letter who also posted it might have intended to help the *sender*, who apparently lost the letter, rather than the *recipient* who might even be unaware of the letter’s existence. In this case, it would be possible that finders simply did not want to help a sender from the German in-group, who had apparently lost a letter intended for a generic member of the stigmatized Turkish out-group. Still, when an in-group member lost a letter addressed to a person holding a doctorate degree, finders may have been motivated to post it to help his or her fellow in-group member communicating with a doctor. We explored this latter notion, which is also in line with predictions derived from views of parochial altruism, in Experiment 2.

We kept the presence of the doctorate degree for the recipient constant in Experiment 2 and designed it to investigate more closely the question of whom finders actually direct their help to, the sender or the recipient of a lost letter.

## Experiment 2

### Design and Procedure

In Experiment 2, we investigated whether the increase in helping behavior toward the competent member of the stigmatized out-group was due to the in-group membership of the sender who always belonged to the German in-group in Experiment 1. We addressed letters to a doctor whose name was either indicative of an in-group recipient (German) or of a recipient belonging to the stigmatized out-group (Turkish). We also varied the name of the *sender* that signaled his group membership (in-group vs. out-group). There were no recipients without doctorate degree in Experiment 2.

In total, *N* = 100 stamped letters were dispersed, *n* = 25 letters per condition in a 2 (sender’s group: in-group vs. out-group) × 2 (recipient’s group: in-group vs. out-group) field study. As per Experiment 1, each letter was attached behind a car’s windshield. All letters included hand-written addresses of recipient and sender. The sender’s address was a local one in Bielefeld, Germany, where the letters were distributed. The names of the recipients were Markus Schäfer (German) and Ali Yildirim (Turkish). The senders were named Fatih Celic (Turkish) or Frank Meier (German). We included a short note in each envelope that was an invitation to a party in case any finder opened the letter. The content of the invitation could not be read through the envelope.

### Results and Discussion

As in Experiment 1, statistical tests based on the resulting 2 × 2 contingency tables are one-tailed. As **Table 3** indicates, fewer letters arrived when the sender was from the stigmatized Turkish out-group rather than from the German in-group, χ^2^(1, *N* = 100) = 4.11, *p* = 0.02, φ = 0.20. Importantly, for the Turkish recipient, significantly less letters were returned when the sender had a Turkish name than when the sender had a German name, χ^2^(1, *N* = 50) = 2.92, *p* = 0.04, φ = 0.24. When the recipient of the letter belonged to the German in-group, this difference was not statistically significant, χ^2^(1, *N* = 50) = 1.33, *p* = 0.12. The overall return rate for the German recipient did not significantly differ from that for the Turkish recipient, χ^2^(1, *N* = 100) = 0.16, *p* = 0.34. This finding is in line with Experiment 1, in which we also did not find any difference in return rates when each of the German and Turkish recipients held a doctorate degree.

**Table 3:** Experiment 2: return rates of letters as function of the recipient’s and the sender’s group membership as written on the lost letters.

	Recipient’s group membership	
	In-group (German)	Stigmatized out-group (Turk)
In-group sender (German)	17 (68%)	17 (68%)
Stigmatized out-group sender (Turk)	13 (52%)	11 (44%)

*Absolute number of returned letters and respective percentages in parentheses per condition. Dispersed letters per cell were n = 25. The recipient’s name was consistently preceded by a doctorate.*

The motivation to facilitate the correspondence between a letter’s sender and a competent member of the stigmatized out-group, or to even make such a communication possible, apparently depends upon the sender’s group membership. That an in-group member seeks to interact with a highly competent member of the stigmatized out-group seems to be a key factor in this attenuation of intergroup discrimination.

## General Discussion

Together with the pilot study, the results from our two field experiments substantiate claims from previous studies, and importantly, also yield novel insights for research on in-group favoritism as one of two aspects of parochial altruism on various levels (also see Dorrough et al., 2015). Firstly, discrimination in favor of one’s in-group over different out-groups apparently depends on the evaluation of these out-groups (cf. Cuddy et al., 2009). In the present research, differences in helping behavior (i.e., posting a letter) directed at in-group and out-group members were only observed when a stigmatized (vs. non-stigmatized) out-group was compared with the in-group. Secondly, the results show that the perceived competence of the respective member of the stigmatized out-group was a moderator of this in-group favoritism; When a member of a stigmatized out-group was perceived as competent (i.e., holding a doctorate degree), he received a number of letters that did not deviate from the amount the in-group member received. Thus, we assumed that an indicator of competence can serve as buffer against discrimination of a stigmatized out-group member under certain conditions (Experiment 1). Thirdly, while our first experiment could not clearly differentiate whether *in-group love* exclusively accounted for the discrimination effect against the generic member of the stigmatized out-group, Experiment 2 revealed the contribution of *out-group spite*. Return rates were particularly low when a member of the stigmatized out-group had ostensibly lost a letter that was addressed to a recipient of the same stigmatized out-group, even though the recipient was highly competent. Thus, it was not only the recipient of the letter *per se* that received help when he was a highly competent individual. Importantly, help in form of posting that letter was granted only when the *sender* who intended to communicate with an out-group doctor belonged to the in-group. This notion is in line with theoretical conceptions of parochial altruism (De Dreu et al., 2014; Rusch, 2014) because it might well be of evolutionary advantage to help in-group members when they intend to interact with highly competent out-group members.

As demonstrated in our pilot study, Germans apparently regard the French as more competent than Turks. To our knowledge, this differentiation in stereotyping between two out-group nationalities has not been reported in the German context yet (cf. Asbrock et al., 2014). Crucially, such differential stereotyping has important implications for research and practice: While previous research mostly focused on identifying stereotypes and stigmatization of one single out-group based on ethnic background (e.g., Lin et al., 2005), the evidence for different degrees or patterns of stereotyping regarding members of different out-group nationalities may enable researchers to articulate more nuanced predictions regarding intergroup perceptions and behavior toward different out-group nationalities and their members.

According to SIT (Tajfel and Turner, 1979), encountering highly competent out-group members might be perceived as threat to the in-group’s high status. Dovidio and Gaertner (1981) found empirical support for this assumption. In their classic investigation on helping behavior in an intergroup setting, they showed that White participants were less helpful toward a Black individual, when the Black person was introduced as supervisor (versus subordinate). The perceived stability of intergroup status hierarchy has consequently been studied as an important factor in providing support for empowerment help toward members of a low-status out-group (Cunningham and Platow, 2007): When in-group members perceive that their group’s superiority over an out-group might become unstable, they show less helping behavior toward members of the out-group than when they perceive stability in the hierarchy in favor of their own group. However, it is rather unlikely that encountering one single competent individual from an otherwise stigmatized out-group represents a serious threat to the stability of an entire society’s socio-economic hierarchy. According to the present results, the letters addressed to a competent out-group member did not lead to a threatened identity, because the return rates of these letters were higher compared to letters addressed to a generic member of the stigmatized out-group. Still, it seems promising to investigate potential underlying processes of parochial altruism linked to threat perceptions (cf. De Dreu et al., 2010), since previous research has provided substantial evidence for the relation of threat and social discrimination (Branscombe et al., 1999).

There is another process that might have influenced our results: In both experiments, all letters contained recipient addresses located in Germany. Accordingly, it might be possible that the superordinate identity of “people living in Germany” became salient, and previous research has demonstrated that a shared identity can explain helping behavior toward members of an out-group (Levine et al., 2005). Independently of a potential salience of a common identity, the present research showed differential return rates depending on the stigma of the out-group. Thus, the results indicate that despite the potential presence of a shared identity via country of residence, differences in helping behavior can be found in a lost letter paradigm.

Many researchers have reasoned that stereotypes make parts of our lives a lot easier (e.g., Macrae and Bodenhausen, 2000). With the indicator of competence, the mechanism that leads to discrimination against members of an otherwise stigmatized out-group was attenuated. It is important to acknowledge that subtyping processes may have contributed to the differences in helping behavior between the members of the stigmatized out-group with versus without the doctorate degree. A subtyping process becomes probable when group members disconfirm a group stereotype. In order to maintain the stereotypes, such *exceptions to the rule* are clustered together and set aside (Maurer et al., 1995). Thus, adding a doctorate degree to the name of the out-group member could have (mis)lead the finders of the letters to believe that the recipient is a rather atypical member of the out-group that is otherwise stigmatized as incompetent.

We argue that, together with the information that a member of the in-group intended to deliver a message to the out-group member, the negative stereotype against the particular out-group was disconfirmed and the positive stereotype about highly competent exemplars guided the finders’ behavior (also see Sinclair and Kunda, 1999). In other contexts, disconfirming a stereotype about an out-group otherwise stigmatized as cold may also lead to an increase in helping behavior. The dimension of warmth also seems to play an important role in providing help for members of an out-group because in-group members may want to deliver a positive, warm picture of their own group (van Leeuwen and Täuber, 2012).

### Limitations

We assumed that the vast majority, if not all, of the dispersed letters were found and posted by members of the German in-group and not by members of the out-groups. Due to the nature of lost-letter studies, we do not have any data to support this assumption. However, we note that if letters were found and posted to a large extent by members of an out-group, this would have worked *against* our hypotheses and the present data patterns. As Turks constitute the largest ethnic minority in Germany, a potential finder that did not belong to the German in-group would have most likely belonged to this largest out-group. These finders would presumably not have discriminated against a generic sender or recipient of their own in-group.

Future replications of the present studies might consider using larger samples, that is, distribute more letters to increase statistical power. Especially in our second field experiment, a larger statistical power may have, for example, revealed a significant difference in letter return rates for the in-group recipient between the out-group-sender versus the in-group-sender conditions.

We note that the return rates of letters were lower in Experiment 2 than in Experiment 1. However, more than the absolute return rates; the relative rates between the experimental groups were of interest for the present research. In a recent investigation of differences between culturally diverse districts in the city of Berlin, Germany, Koopmans and Veit (2014) have shown that return rates can vary very strongly even across neighborhoods, namely, between 32 and 88% in their study.

## Conclusion

Assumptions about deficient competence in members of stigmatized out-groups can lead to subtle forms of intergroup discrimination. One potential practical implication of the present research is that highly competent exemplars of stigmatized out-groups should not be presented as outstanding, incidental, and atypical instances of this out-group in order to overcome simple subtyping (see Maurer et al., 1995). Instead, interactions between in-group members and competent individuals from stigmatized out-groups should be treated as typical and regular instances. For example, on television programs, members of the in-group could interview particularly competent experts who are members of an otherwise stigmatized out-group. This procedure could be a promising and highly non-reactive avenue in promoting intergroup acceptance by refuting subtle yet socially shared stereotypes against minority out-groups. Future research should explore effects of such media exposure on diminishing intergroup discrimination.

## Author Contributions

JH, AB, JR, and DH planned the research. JH, AB, and JR conducted the studies. JH and AB analyzed the data. JH, AB, and JR drafted the manuscript. DH provided comments and revisions. All authors approved the submission of the final version of this manuscript.

## Conflict of Interest Statement

The authors declare that the research was conducted in the absence of any commercial or financial relationships that could be construed as a potential conflict of interest.
